# Biomass Productivity and Photosynthetic Activity in *Ulva compressa* (Chlorophyta) in Raceway Photobioreactors Under Stress Conditions

**DOI:** 10.3390/plants13213038

**Published:** 2024-10-30

**Authors:** Victor Robles-Carnero, Rafael Sesmero, Felix L. Figueroa

**Affiliations:** 1 Andalusian Institute of Biotechnology and Blue Development (IBYDA), Experimental Centre Grice Hutchinson, University of Malaga, Loma de San Julián, 2, 29004 Malaga, Spain; sesmero@uma.es (R.S.); felixlfigueroa@uma.es (F.L.F.); 2 Faculty of Sciences, Department of Botany and Plant Physiology, Campus de Teatinos, University of Malaga, 20071 Malaga, Spain

**Keywords:** acclimatization, algal culture density, photosynthetic activity, raceway photobioreactor, *Ulva compressa*, stress conditions

## Abstract

Research in seaweed cultivation technologies aims to increase production and reduce costs, leading to more efficient and sustainable processes. In this study, we analyzed the outdoor production of *Ulva compressa* cultured in summertime at different stocking densities of 0.6, 0.8 and 1.0 kg Fresh weight (FW) m^−2^ in a raceway photobioreactor with 30 m^2^ surface (3000 L), and its relation to photosynthetic activity. Under the experimental conditions of high temperature (>28–30 °C) and pH > 9 in culture water, higher seaweed density resulted in lower specific growth rate. The biomass production has been related to photosynthetic activity by using in vivo chlorophyll a fluorescence. Dynamic photoinhibition was observed at noon, which was less severe in cultures with higher algal densities. However, photosynthesis recovered in the afternoon. Seaweeds that were acclimatized for a week to the conditions of 1.0 kg FW m^−2^ stocking density showed an increase in biomass growth and absence of photoinhibition compared to non-acclimatized thalli. In conclusion, the cultivation of *U. compressa* in a mid-scale raceway photobiorreactor under conditions of high irradiance and temperature and low nutrient input, exhibited the best photosynthetic performance and hence the highest growth rates for the highest culture density assayed (1.0 kg FW m^−2^).

## 1. Introduction

Macroalgae provide a multitude of ecosystem services for the following categories; e.g., regulation (carbon fixation, pH increase, biofiltration), supporting (habitat, biodiversity, photosynthesis), cultural (science and education) and supply (food, feed, drugs, fibers, etc.) [1,2]. Indeed, seaweeds are gaining interest in the scientific community, industry and society in general for their potential as a source of valuable, sustainable biomass in the food, feed, chemical and pharmaceutical industries; but also for its interest as biofilters, water quality indicators and other biotechnological applications [3,4,5,6,7].

The aquaculture industry has developed some strategies for cultivating marine organisms, including macroalgae, to increase biomass yields and reduce pollutant release [8,9]. Seaweed aquaculture provides ecosystem services that can improve conditions in coastal waters for the benefit of other living organisms and the environment [10].

The cultivation of seaweeds in open sea systems has advantages compared to land-based systems related to productivity, scaling-up and costs. However, sometimes safety issues arise due to the presence of chemical and biological contaminants [11]. Algae biomass coming from harvesting in the natural environment or from aquaculture production at sea could present variability in terms of its quality with respect to the adsorption and absorption of heavy metals from the water that accumulate in the biomass [12,13], especially in areas close to big cities and to mining activity.

Land-based aquaculture can also have environmental impacts, particularly if high levels of chemical fertilizers are dumped into the sea, leading to eutrophication. Open systems are more susceptible to environmental contamination from pollutants and unwanted species, while controlling parameters such as temperature and pH can be more challenging. On the other hand, closed systems allow for greater control of parameters such as nutrients and reduce both contamination of the growing medium and the presence of unwanted species, while pH and temperature maintenance is more expensive. These systems offer the possibility of controlling certain production-related traits, such as nutrient concentration, light quantity and quality, and algae concentration [14].

Therefore, solutions based on the cultivation of algae species in land-based systems in which main cultivation parameters are controlled, make this type of system very interesting for the continuous production of standard-quality biomass to obtain high-value biocompounds [15,16]. Species of the genus *Ulva* have been proposed as a valuable resource for a long time due to their multiple uses and high growth rates [17,18,19]. This genus has been used as efficient biofilters in integrated multitrophic aquaculture (IMTA) systems by using fishpond effluents [20,21]. The importance of this genus has led to the establishment of a European Cost Action (CA20106—Tomorrow’s “wheat of the sea”: *Ulva* a model for an innovative mariculture) focused on its research [22].

The present work aimed to achieve progress towards the cultivation of the macroalgae *Ulva compressa* in well-known systems such as raceway ponds, which were previously assayed in various microalgae species [23,24,25]. This system is based on the closed recirculation of water and nutrients, which optimizes the use of these resources as well as the energy consumption.

Specifically, we evaluated the optimal algae density in terms of maximum productivity in this outdoor culture system under non-controlled environmental factors. Lower nutrient inputs, compared to other studies [26], were chosen in order to evaluate the ability of this algae to reach optimal productivity, with minimum inputs for the sake of a more sustainable and economic culture. Nevertheless, the nutrient content is within the range of previous studies using IMTA systems [27,28].

The experiments were carried out in Southern Spain within the summer period, reaching a high temperature in the water (>30 °C) and daily irradiance of photosynthetic active radiation (PAR, λ = 400–700 nm) higher than 12,000 kJ m^−2^ [29]. The ability of *Ulva* to live and grow above this water temperature has been previously demonstrated [30,31], but there is lack of evidence about the effect of the algae density on the physiology and productivity in this culture system, especially the effect of high both dissolved oxygen and pH due to high photosynthetic capacity. It is known that *Ulva* spp. can survive in supralittoral ponds under high temperature and pH, in high-salinity conditions caused by water evaporation, and in low-salinity conditions [32,33]. *Ulva* spp. present highly efficient different enzymatic systems for bicarbonate assimilation under high pH [34,35].

Once the optimal thalli density was determined, we also analysed the effect of acclimatization to these environmental conditions. Growth rate is related to the photosynthetic activity estimated by using in vivo chlorophyll *a* fluorescence of photosystem II (PAM fluorometers) [36,37]. This technique has been proven as good tool in seaweeds to evaluate both photosynthetic efficiency and capacity through maximal quantum yield (Fv/Fm) as an indicator of photoinhibition and Electron Transport Rate (ETR) as an estimator of photosynthetic capacity (gross photosynthesis), respectively [36,37,38]. Good correlation between gross photosynthesis and ETR has been found both in green micro and macroalgae [27,36,39,40]. In situ monitoring of effective quantum yield under solar radiation allowed us to determine the daily productivity under different environmental conditions of *Ulva* spp. growing in tanks [27,39,41].

## 2. Results

### 2.1. Diurnal Variation in Water’s Physical and Chemical Variables During the Course of the Culture

**Figure 1:**
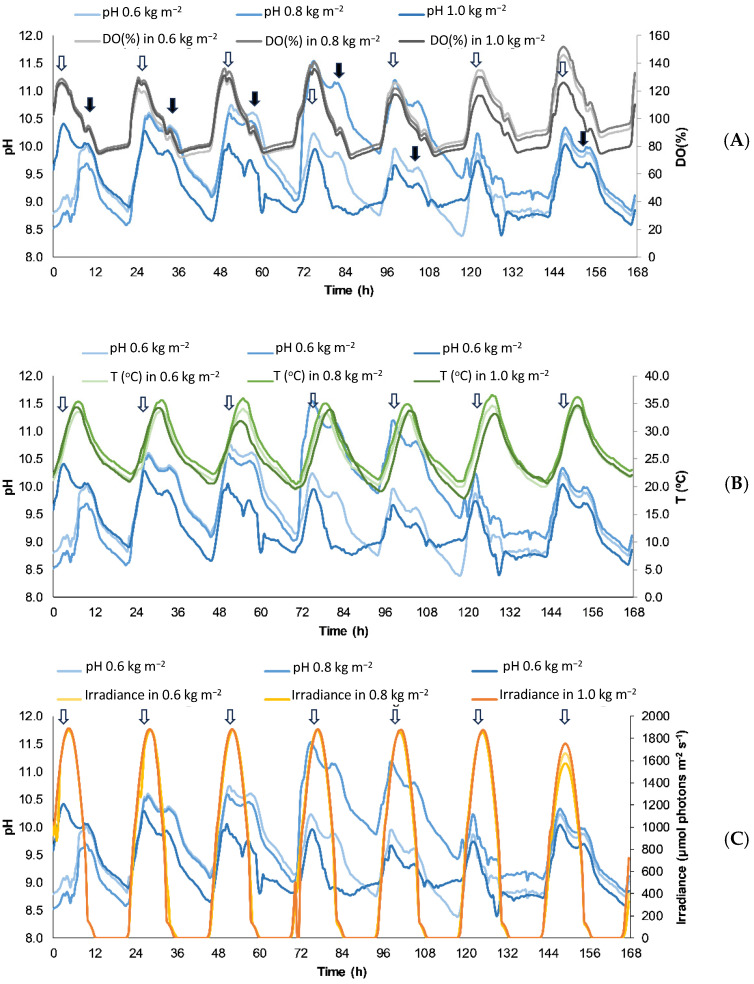
pH vs. DO(%) (**A**), pH vs. irradiance (**B**) and pH vs. temperature (**C**) patterns at the raceway system during the experiments with different Ulva compressa culture densities (0.6, 0.8 and 1.0 kg FW m^−2^). Time 0 in X-axis corresponds to 9.00 a.m. Open arrows correspond to midday; bold arrows indicate transitory pH increases in the afternoon.

The correspondence of pH with the rest of the variables is shown in Figure 1.

The pH and dissolved oxygen (DO) increased from the morning to midday reaching the maximum values just before noon (Figure 1A). This pattern must be related to a positive net photosynthesis. Depending on the biomass density, different patterns are observed on day 4. At the lowest density (0.6 kg fresh weight (FW) m^−2^) and the highest (1.0 kg FW m^−2^) a drop in pH values was observed, which implies a reduction in net photosynthesis; while in the density of 0.8 kg FW m^−2^ an increase in pH was observed on day 4, which would mean an increase in the net photosynthesis of the system, but a decrease was observed later.

Around midday, a sharp decrease in pH and DO was found at temperatures around 27 °C (Figure 1B). This indicates that high irradiance induces a reduction of electron transport rate as an estimator of gross photosynthesis, possibly due to photoinhibition (Figure 1C), whereas high temperatures increase respiration and reduce oxygen solubility in water. The drop in DO at noon was produced after the maximal irradiance and coupled to the maximal temperature. Interestingly, maximal temperature was produced 1–2 h after the maximal irradiance (Figure 1), then in the early afternoon recovery of DO was produced. A small, transitory increase in pH was observed (bold arrows in Figure 1A), that implies a positive net photosynthesis. This transitory peak in photosynthesis induces a reduction of the negative slope in DO pattern.

### 2.2. Biomass Growth Rate and Nutrient Assimilation in the Raceway Ponds

For 0.6, 0.8 and 1.0 kg FW m^−2^ densities, biomass growth rate values at FW basis were 36.7, 39.1, 38.1 g FW m^−2^ day^−1^, whereas at Dry weight (DW) basis were 6.23, 6.64, 6.48 g DW m^−2^ day^−1^, respectively (Table 1).

Specific Growth Rates (SGRs) of 5.94, 4.90 and 4.07% day^−1^ were found for the mentioned culture densities in our study (Table 1). Biomass FW increments were 7.7, 8.2 and 8.3 kg FW week^−1^ for the 0.6, 0.8 and 1.0 kg FW m^−2^ densities, respectively (Table 1).

When normalizing biomass increment to nutrient consumption (i.e., yield per N and P), 0.611, 0.651 and 0.659 kg FW biomass mg^−^^1^ N, 8.280, 8.817 and 8.925 kg FW biomass mg^−^^1^ P was produced, as can be observed in Table 1 at 0.6, 0.8 and 1.0 kg FW m^−^^2^ biomass concentration, respectively.

Regarding nutrient assimilation, 900 µmol L^−^^1^ of nitrogen and 54 µmol L^−^^1^ of phosphate was injected weekly in the culture and the nutrient concentration always was depleted in the water; i.e., the nutrient uptake efficiency (NUE) reached values of 100% in every algal density. Nutrient uptake rate (NUR) values were 5.952, 4.464 and 3.571 µmol N g DW^−1^ h^−1^ in 0.6, 0.8 and 1.0 kg FW m^−2^, respectively.

### 2.3. Physiological and Functional Variables in Cultures Under Different Algal Density

#### 2.3.1. In Situ Photosynthetic Activity

**Figure 2:**
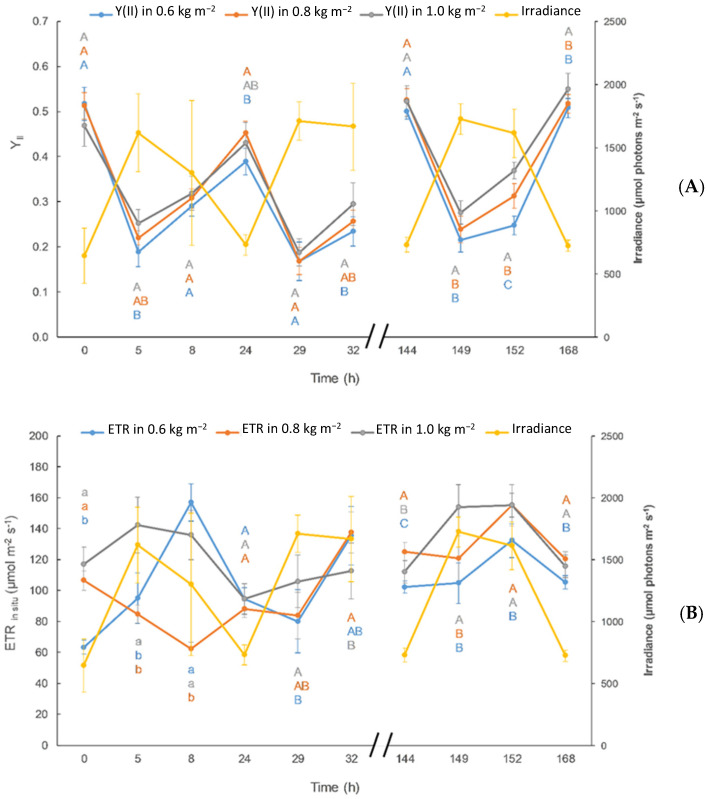
Effective quantum yield (Y_II_) (**A**) and in situ Electron Transport Rate (ETR_in situ_) (**B**) expressed in µmol electron m^−2^ s^−1^ measured in Ulva compressa growing in the raceway pond during the experiments at different culture density. Average irradiance level is also represented in the secondary Y-axis. Different letters correspond to significant differences between treatments at a fixed time following one-way ANOVA (*p* < 0.05) after Tukey’s test; lowercase letters correspond to non-parametric Kruskal-Wallis test.

As shown in Figure 2A, there was a gradual rise in the value of Y_II_ except 24 h after starting the experiment, when a pronounced fall was observed. This indicates a stress of the algae since the algae were transferred from tanks at a density of 30 g FW L^−1^; i.e., from a shade environment in the tanks to a raceway with higher solar radiation exposure. As in the case of DO (Figure 1A), a decrease of ETR values was observed at noon under maximal irradiances. Thus, if we consider ETR as an estimator of photosynthetic capacity and biomass productivity [39,40], this result may be indicative of a decay in biomass productivity due to an increase in photorespiration and also to the presence of photoinhibition by excess of oxygen, which may include production of radical oxygen substances.

#### 2.3.2. Ex Situ Photosynthetic Activity: Rapid Light Curves (RLC)

Maximal quantum yield (F_v_/F_m_) was higher under the highest algal density than that under 0.6–0.8 kg FW m^−2^ (Table 2).

The photosynthetic efficiency (α_ETR_) was not significantly different among the different algal densities. However, the saturation irradiance (E_k_) increased in cultures under 1.0 kg FW m^−2^ density in spite of the self-shading.

Maximal non photochemical quenching (NPQ_max_) was higher under 1.0 kg FW m^−2^ density compared to lower algal densities, while ETR_max_/NPQ_max_ decreased under 1.0 kg FW m^−2^ algal density cultures compared to 0.6 kg FW m^−2^.

Maximal ETR under outdoor raceway ponds (ETR_in situ_) reached values of 150–160 µmol electrons m^−2^ s^−1^, whereas ex situ ETRmax reached values of 90–94 µmol electrons m^−2^ s^−1^.

### 2.4. Physiological and Functional Variables in Acclimatized Thalli

Acclimatized algae coming from 1.0 kg FW m^−2^ stocking density (as explained in Section 4.2) showed an improvement in growth-related parameters (Table 3). SGR and biomass increment raised 0.7% day^−1^ and 1.7 kg FW week^−1^, respectively. This biomass increment corresponds to a 20.8% in 1 week compared with the non-acclimatized algae. Improvements in the efficiency in nitrogen and phosphorus assimilation were also found, from 0.66 to 0.79 kg FW mg^−1^ N and 8.93 to 10.75 kg FW mg^−1^ P.

**Figure 3:**
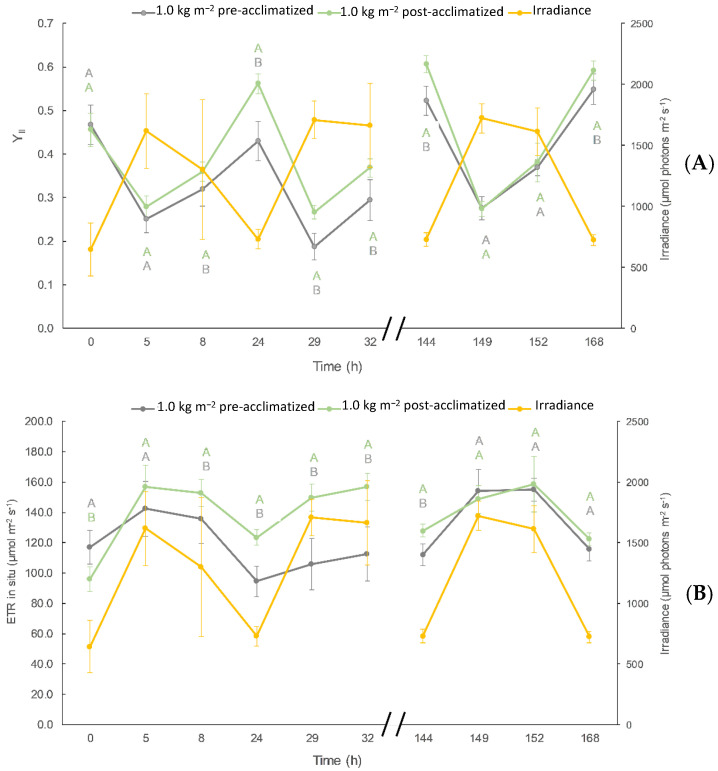
Y_II_ (**A**) and ETR_in situ_ expressed as µmol electron m^−2^ s^−1^ (**B**) in non-acclimatized and acclimatized Ulva compressa cultures at 1.0 kg FW m^−2^ density. Average irradiance level is also represented in secondary Y-axis. Different letters correspond to significant differences between treatments at a fixed time following—Student’s *t*-test (*p* < 0.05).

Photosynthetic activity, expressed as in situ Y_II_ and ETR, were higher in acclimatized- than in non-acclimatized algae (Figure 3). Both photosynthetic parameters improved, especially between 8 h and 32 h after the beginning of the culture.

All photosynthetic parameters determined by in vivo Chl a fluorescence increased in acclimatized algae (Table 4). The algae present a metabolism of sun type algae (increased E_k_ and ETRmax), revealing its higher photosynthetic capacity. The NPQ_max_ decreased in acclimatized algae. On the other hand, as expected, the ETRmax/NPQmax increased.

Many research studies on *Ulva* spp. production have been conducted, as shown in Table 5 (modified from [26]). Different algal densities, variety of nutrient source, different tank volumes, and number of water exchanges have been studied. Our study makes use of chemical fertilizers under low concentration compared to other works and with no water exchanges. Table 5 shows the high variability in the growth rate results obtained in our work and in previous studies from different authors. This is due to the diversity of tanks and volumes, etc. used in each study. A comparison of biomass productivity expressed as g DW m^−2^ day^−1^, reveals significant discrepancies between studies. However, when these data are converted into g L^−1^ day^−1^, the differences are less pronounced.

### 2.5. Functional Relationship Between Variables

Values of Pearson correlation between different functional variables are shown in Table 6. A correlation was observed between SGR and nitrate uptake rate (NUR), as well as between NUR and the ETRmax/NPQmax ratio. However, no correlation was found between SGR or NUR and ETRmax.

## 3. Discussion

### 3.1. Physical and Chemical Variables During the Culture

The pH, DO and temperature presented variations depending on the concentration of biomass in the reactor and the time of day (Figure 1). ETR decreased close to noon under the highest pH and DO values. This decrease of ETR can be related to photoinhibition. Due to recovery of ETR values in the afternoon, it can be considered dynamic photoinhibition. The phenomenon of dynamic photoinhibition, characterized by a decline in Fv/Fm followed by a subsequent recovery, has been observed in contrast to the more prolonged and irreversible process of chronic photoinhibition, which occurs without a return to normal levels [52,53,54].

In the afternoon, with the decrease in incident solar radiation (between 1200–1300 µmol photons m^−2^s^−1^), *Ulva compressa* can use bicarbonate due to high activity of carbonic anhydrases [34]. Nevertheless, it has been demonstrated that CO_2_ addition improved photosynthetic activity and increased productivity of *Ulva*, provided that pH does not rise above 11–12 [55]. In this study, in the periods with high pH (>9) (Figure 1A–C), bicarbonate must be the main source of carbon, and it is transformed to CO_2_ by the action of carbonic anhydrase enzymes. Thus, the efficient use of bicarbonate is an advantage because it reduces the costs (since the expense of CO_2_ is one of the limitations in many algal cultures), avoiding risk of contamination by other algae that cannot grow at such high pH [34]. Limitations in access to carbon (in the form of CO_2_ or bicarbonate) cause a substantial reduction in the photosynthesis of the algae and reduce its productivity, giving rise to a negative net photosynthesis and a prevailing respiratory process [56]. Similarly, we found that both pH and DO in the raceway decrease even during the hours of high radiation, although later at sunset the pH temporarily recovers, to fall again later during night (Figure 1A).

### 3.2. Effect of Algal Density on Biomass Growth Rate and Nutrient Assimilation

The SGR decreases as the density of our culture increases (Table 1). Similar results were obtained in other studies [6,16,26], where tanks were used as culture systems at higher algal density than in our work (1.0–3.0 kg FW m^−2^). The authors of [17] conducted a screening study on 48 strains of *Ulva compressa.* and found an SGR average of between 3 and 6% day^−1^, with *U. pseudorotundata* being one of the species with the highest SGR together with *Ulva prolifera*. Considering the night period, the average SGR was 8.63% but certain *Ulva* strains can reach a specific growth rate of 12–16% period^−1^ [17]. The diversity in the amino acid content among the strains was also very high. Thus, it is crucial to investigate the response of different strains in terms of growth and biochemical contents at similar culture conditions in order to optimize the productivity of high-value biocompounds, as has been proven in diverse screening studies, mainly for food and cosmeceutical applications [57,58,59,60].

In contrast with SGR, biomass FW increased at higher initial densities, ranging from 7.7 to 8.3 kg FW week^−1^. This is not a remarkable increase of production, but under the highest initial biomass of *Ulva*, less competition with microalgae in the reactor was visually observed compared to lower initial densities. Therefore, the higher the initial density, the less competition for nutrients was taking place. When normalizing biomass increment to nutrient consumption; i.e., yield per N, cultures with higher density presented higher productivity yield, indicating a more efficient use of nutrients; i.e., more biomass was produced with less nutrient consumption (Table 1).

As mentioned above, algal density showed an inverse correlation with SGR, but the highest density presented the highest total production under our experimental conditions. In the present work, a low concentration of nutrients was used compared to other studies; i.e., a total of 0.06 mg N m^−2^ day^−1^ of nitrogen input, much lower than in other studies (e.g., [26] used 0.81–0.99 g N m^−2^ day^−1^). Still, these nutrient concentrations are much higher than that in the coastal Mediterranean waters [61]. In any case, the content of inorganic nitrogen used in this study is in the range of the nitrogen levels in the fishpond effluents under IMTA [27,28,62,63]. Thus, if increasing nutrient input, an increase in biomass productivity could be expected.

Values of NUE and NUR found in our work were similar to other studies [5] (NUE 100% in every algal density, NUR values of 5.952, 4.464 and 3.571 µmol N g DW^−1^ h^−1^ in 0.6, 0.8 and 1.0 kg FW m^−2^, respectively).

### 3.3. Effect of Algal Density on Physiological and Functional Variables

As in the case of DO (Figure 1A), a decrease of ETR values was observed at noon under maximal irradiances. Thus, if we consider ETR as an estimator of photosynthetic capacity and biomass productivity [39,40], this result indicates a decay of the photosynthesis that could be related to photoinhibition by decay of chlorophyll by excess of light. This was a transient response since recovery of ETR values were observed in the afternoon. Recovery at different times of the day was observed as a consequence of the algae acclimatization to the new culture conditions. Dynamic photoinhibition is regarded as a physiological strategy of photoprotection, and temporary decrease of effective quantum yield could be a simple matter of zeaxanthin-induced fluorescence quenching; i.e., increase in YNPQ.

Under the highest culture density, the lowest photoinhibition in the central hours of the day (5, 29 and 149 h) is found to be a consequence of the self-shading and subsequent photoprotection pattern, as reported by [52].

A pronounced drop in ETR_in situ_ values within 24 h from the beginning of the experiment in all densities assayed is observed (Figure 2). This decrease could be related to stress due to excess of radiation, as obtained in other studies for *Ulva* under different physiological conditions [27,41].

The positive correlation between in situ maximal quantum yield (F_v_/F_m_) and algal density (Table 2) confirmed photoprotection against photoinhibition by self-shading of the thalli in the ponds, as reported in [52]. The ETR_max_ was similar among the different algal densities, reaching values higher than that reported in *U. pseudorotundata* growing in tanks, moving with an air pump and fed with fishpond effluents [6].

The ex situ photosynthetic activity revealed that, under the three algal densities assayed, the E_k_ values corresponded to sun-type algae, as it has been reported in other *Ulva* species growing in tanks [27,64]. Sun-type algae (i.e., intertidal algae) presented higher ETR and E_k,_ and lower α_ETR_ than algae from shade habitats (subtidal algae) [36,65].

High NPQ (Table 2) indicates high capacity for energy dissipation. This photosynthetic parameter is an indicator of optimal photoprotection, since NPQ is the ratio between two yield losses; namely, Y_NPQ_ and Y_NO_, with Y_NPQ_ being a mechanism related to the dissipation of energy as heat and fluorescence through photoregulated mechanisms (i.e., xanthophyll cycle) and Y_NO_ is passive dissipation [66]. High values of Y_NPQ_ than Y_NO_ is an indicator of optimal photoprotection under high irradiance or other stress on photosynthesis, such as high temperature or increased UV radiation [67,68]. The ETR_max_/NPQ_max_ ratio has been used as a physiological indicator expecting to be higher under optimal growth conditions; namely, when production is greater than energy dissipation. Conversely, this ratio is anticipated to decrease under increased UV radiation [67] or under acidification and low nitrogen conditions [27]. However, in this study the ETR_max_/NPQ_max_ decreased under 1.0 kg FW m^−2^ algal density cultures compared to 0.6 kg FW m^−2^. Under 1.0 kg FW m^−2^ it seems like energy dissipation or yield losses are higher to acclimatize to the environmental conditions; i.e., nutrient competition under high solar exposure. Nevertheless, despite the comparable ETR_max_ and diminished ETR_max_/NPQ_max_ in 1.0 kg FW m^−2^ relative to the 0.6 kg FW m^−2^ alga density, the biomass yield remained consistently high.

Higher values of in situ photosynthetic rate compared to ex situ, both by fluorescence determination (ETR) and oxygen evolution (gross photosynthesis) has been previously reported in *Ulva* spp. [69] and other micro and macroalgal species [39,40]. High correlation between ETR and gross photosynthesis has been reported in *Ulva* species and thus ETR has been demonstrated to be a good indicator of gross photosynthesis [27,36,70]. The highest in situ ETR compared to ex situ has been explained due to the incident actinic light. Under the in situ condition, algae are exposed to solar radiation and under ex situ measurements the actinic light presents a narrow spectrum (red light); thus, a broader range of quanta absorbed by accessory pigments are available to conduct photosynthesis under in situ compared to ex situ [27,39]. In addition, under laboratory conditions, a unique piece of thallus is used to conduct the RLC and although three replicates are conducted, each thallus was continuously exposed to increasing irradiance for several minutes. Under these conditions, acclimatization to light can be performed and consequently Y_II_ can be reduced. In contrast, under ex situ measurements, eight different thalli randomly selected in the culture were used, and there is no exposure to increasing irradiances as there is when performing the RLC [27,39].

### 3.4. Effect of Algal Acclimatization on Physiological and Functional Variables

The improvement in growth parameters observed (Table 3) is due to an increase in photosynthetic capacity (Figure 3) by acclimatization to the stressful processes of the culture medium, such as temperature, light intensity, as well as nutrient deficiency in the culture medium. The afternoon recovery; i.e., increase of Y_II_ (from 5 h to 8 h, corresponding to 2.00 p.m. and 5.00 p.m., respectively) was higher in acclimatized algae, which contributed to higher ETR_in situ_, indicating that more energy can be available for growth [37].

As we explained in point 2.4, the algae presented a sun-type metabolism (high E*k* and ETR_max_). The reduction of NPQ_max_ indicates that the algae require less energy dissipation because of physiological acclimatization to the high irradiance. The increment of ETR_max_/NPQ_max_ indicates that algae have more energy available for growth, as has been previously reported under optimal growth conditions in other seaweeds including *Ulva* spp. [27,39,67].

### 3.5. Functional Relationship Between Variables

SGR correlated to nitrate uptake rate (NUR) since photosynthetic productivity is related to the availability and assimilation rate of nitrate [39]. NUR correlated to ETR_max_/NPQ_max_ but not with ETR_max_, indicating that the nitrate assimilation rate and specific growth is more related to the ratio between productivity and energy dissipation than with only productivity. If the growth is represented as specific growth rate in % d^−1^, a positive correlation with ETR_max_/NPQ_max_ was found, i.e., highest SGR and ETR_max_/NPQ_max_ at 0.6 kg FW m^−2^ (see Table 6).

The data shown in this study have been obtained in the month with the highest irradiance and temperature of the year in Malaga, demonstrating that *U. compressa* can be cultivated in this type of photobioreactor under high solar exposure. Under these conditions, more energy is available for photosynthesis but also, under very high temperature, there is an increase in respiration and, consequently, a reduction in net photosynthesis is to be expected although gross photosynthesis (NP + R) could be less affected by the increase of the eventual respiration. Good correlation between gross photosynthesis measured by oxygen evolution and electron transport rate has been found in *Ulva* spp. [36,70]. High respiration can provide additional ATP to maintain both inorganic carbon and nitrogen assimilation and, in addition, to contribute to photoprotection. On the other hand, high oxygen consumption due to Mehler reaction is expected under the environmental condition in this study [36]. This capacity to live and grow at water temperatures above 30 °C of different *Ulva* strains [30,31], implies its potential as a future cultivated algae for multiple uses in a climate change scenario, in which higher temperatures are expected in southern European countries, such as Spain. In previous works, an induced increase in temperature of 4 °C, which represent a pessimistic scenario for climate change at the end of this century, was found to result in an increase in both gross photosynthesis and ETR_max_ in *Ulva rigida* [27]. However, the observed increase in both photosynthetic parameters was produced only under high nitrate conditions, at both low and high carbon levels. Conversely, the decrease was produced under low nitrogen and high CO_2_ conditions (acidification). Therefore, it can be concluded that the adaptation of *Ulva* to climate change is not solely dependent on temperature, but also on the levels of nitrate and CO_2_ present in the water.

## 4. Materials and Methods

### 4.1. Sampling

The green alga *Ulva compressa* (also called *mutabilis*), (Linnaeus, 1753) used in this study was collected in salt marsh areas of the bay of Cádiz (36°30′ N, 6°10′ W). The algae were transported in thermal boxes to the Grice Hutchinson Research Centre at Malaga University, where the experiments were conducted.

Before the establishment of the culture in raceway systems, thalli of *Ulva compressa* were maintained at stocking densities of 30–50 g L^−1^ outdoor in 300 L square tanks with continuous aeration, in reactors with artificial seawater prepared with unrefined sea salt (Salinas San José (Chiclana, Cadiz, Spain), RSI 24.371/CA) added to tap water of Malaga city reaching salt concentrations of 35 psu. The concentration of main ions was determined by ion chromatography equipment; METROHM 883 Basic IC plus + Autosampler supplied by Methrom AG (Germany). Concentrations of main ions expressed in mg L^−1^ were: (1) Anions: Cl^−^ 19,316.5, Br^−^ 8.6, F^−^ 0.32, SO_4_^2−^ 751.45, NO_3_^−^ 13.0, NO_2_^−^ no detected and PO_4_^3−^ no detected and (2) Cations: Na^+^ 13379.7, Mg^2+^ 215.1, Ca^2+^ 102.1, NH_4_^+^ no detected and K^+^ 146.7).

### 4.2. Experimental Conditions

Experiments were conducted during June–July 2022. For the evaluation of algae density, stocked thalli were transferred from the stocking tanks to a raceway pond of 30 m^2^ surface area and 10 cm depth, i.e., a volume of 3000 L with the above described artificial seawater. 150 µmol L^−1^ NH_4_NO_3_ and 18 µmol L^−1^ KH_2_PO_4_ were added to the reactor 3 times per week by means of agricultural granular fertilizers of the brand Fertiberia^®^ (34.5% N purity) and Fenasa^®^ (22.7% P purity), respectively. These nutrient values are in the range used in IMTA systems in other works [34,70,71]. The algae were grown unattached and kept in movement by the paddlewheel of the raceway reactor. A continuous aeration in the sump was blown without addition of CO_2_.

**Figure 4:**
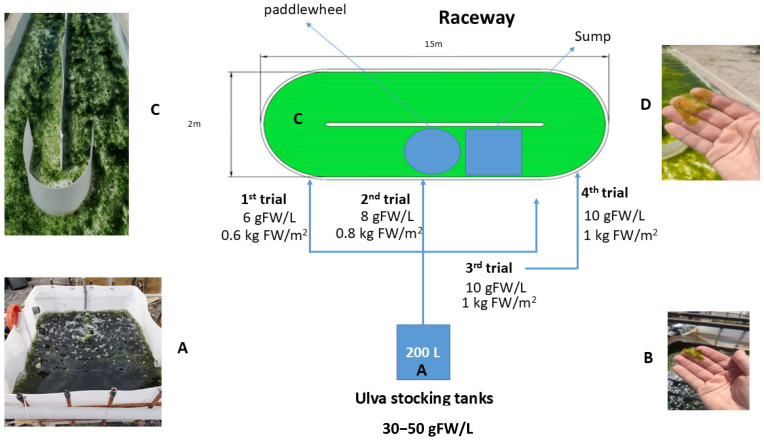
Diagram showing the cultivation system used and the morphological changes observed in the algae. (**A**): stocking tank; (**B**): algae from the stocking tank; (**C**): raceway reactor with the algae in culture; (**D**): algae from the raceway reactor (note the change in size and colour).

As explained in Figure 4, thalli were grown for 7 days in each treatment, which consisted of different thalli densities (0.6, 0.8 and 1.0 kg FW m^−2^). In all treatments, the input biomass came from the same 300 L stocking tanks. Densities were tested sequentially using the same raceway system; i.e., first week at 0.6 kg FW m^−2^, second week at 0.8 kg FW m^−2^ and third week at 1.0 kg FW m^−2^. At the end of each treatment (day 7), the algae were completely collected and growth variables (specific growth rate, biomass increase and N consumption) were measured. The new density treatment was then set up and the same procedure was carried out.

Physico-chemical parameters of the culture (pH, dissolved oxygen (DO) and water temperature, as well as incident irradiance) were monitored throughout the experiments in each treatment (see Section 2.3), as well as physiological and functional variables (photosynthesis, as in vivo chlorophyll a fluorescence-related parameters, as described in Section 2.4).

In order to evaluate physiological changes and growth patterns after acclimatization, a further experiment was carried out at 1.0 kg FW m^−2^ density; algae tested at this density were collected from the raceway reactor at the end of the treatment (1 week) and re-established at the same initial density of 1.0 kg FW m^−2^ after measuring growth-related variables. The culture was then continued for an additional week, after which physiological and growth-related parameters were measured using the same procedure as in the previous test. For the post-acclimatization treatment, the input biomass from the pre-acclimatized 1.0 kg FW m^−2^ density collected at the end of this treatment was used (Figure 4).

### 4.3. Water Physical and Chemical Analysis

Water temperature (T), pH and DO were monitored automatically every 30 min along the experiments using raceway-integrated sensors (Jumo tecLine HD, Jumo Instrument Co. Ltd., UK). Irradiance was measured automatically every 30 min outside the tank as incident irradiance, using HOBO pendant^®^ Light Data Logger (Onset, MA, USA). In order to verify the automatic measurements, water T, pH, salinity and DO were measured 3 times every day manually using portable devices (LAQUAact pH, Horiba Ltd., Kyoto, Japan; EC120, Horiba Ltd., Kyoto, Japan and HI-98193, Hanna Instruments Inc., Woonsocket, USA, Dissolved Oxygen, respectively) at 9:00, 12:00 and 15:00.

To determine changes in concentration of N−NH_4_^+^, N−NO_3_^−^ and PO_4_^3−^, water samples were taken before addition of nutrients and then it was analysed colorimetrically using a continuous flow automated analyser (Technicon AA-2), following the procedure of [72].

The reduction in nitrogen concentration between the time intervals is expressed as percentage and defined as “nutrient uptake efficiency” (NUE) and was calculated by Equation (1) assessing the changes in total nitrogen concentration:

NUE (%) = 100 − [C_t+1_ × 100/C_t_] (1)

where C_t_ represents the initial concentration of nutrients and C_t+1_ represents the concentration after t + 1.

The amount of nitrogen removed per unit of time per volume by seaweed dry weight represent the “nutrient uptake rate” (NUR) and is determined from changes in nitrogen, according to Equation (2):

NUR (µmol N g^−1^ DWh^−1^) = [(C_t_ × V_t_)−(C_t+1_ × V_t+1_)]/(B × Δ_t_) (2)

where C_t_ represents the initial concentration of nitrogen, V_t_ represents the initial volume of the photobioreactor (in L), C_t+1_ represent the concentration of nitrogen after t + 1, V_t+1_ represent the volume of the photobioreactor after t + 1 (in L); B represents dry biomass used initially (g), and Δt represents the time interval between t and t + 1 in hours.

### 4.4. Biomass Growth Parameters and Physiological Variables Measurements

For every assayed density, growth parameters were determined at the end of every experiment (as explained in Section 2.2) by measuring the increment in algal fresh biomass after harvesting. Thalli were collected with a net and manually pressed always by the same person in order to drain out the excess water.

Biomass growth rate parameters were calculated as the biomass increment in fresh weight (FW) or dry weight (DW) basis normalized to units of volume or area. The Specific Growth Rate (SGR) was calculated following the equation:

SGR = 100 × [ln (W_f_/W_0_)]/t (3)

where W_0_ = initial biomass, W_f_ = final biomass, and t expresses the days of culture in the experimental set (adapted from [26])

Relative biomass increment was measured using the equation:

Relative biomass increment = (W_f_ − W_0_)/W_f_ (4)

where W_0_ = initial biomass, W_f_ = final biomass

Photosynthetic activity was estimated through the in vivo chlorophyll *a* fluorescence associated to photosystem II (PSII) by using a Mini-PAM-II fluorometer (Walz GmbH, Effeltrich, Germany), with red light as measuring, actinic and saturating pulse light for both in situ in the raceway ponds and ex situ in the laboratory measurements.

(a) In situ-effective quantum yield (Y_II_) (Equation (5)) was determined outdoors in algae growing in the raceway pond. Y_II_ was used for the determination of Electron Transport Rate (ETR) (Equation (6)) as an estimator of photosynthetic capacity (gross photosynthesis) and algal productivity [40]. Y_II_ was determined as follows: firstly, fluorescence at steady state (F_t_) was calculated by measuring red light and then saturating light pulse was applied (800 ms, 5000 µmol m^−2^ s^−1^) to algal thalli in the reactor to determine maximal fluorescence at light−acclimated samples (F’_m_), according to [27]. Effective quantum yield was calculated as:

Y_II_ = (F’_m_ − F_t_)/F’_m_ (5)

ETR_in situ_ was calculated as:

ETR_in situ_ = Y_II_ × E_PAR_ × A × F_II_ (6)

where E_PAR_ is the irradiance of photosynthetic active radiation (λ = 400–700 nm) of the incident light at the surface of the reactor; A is the absorptance, which was measured every day according to [68]; F_II_ is the fraction of chlorophyll *a* associated to PSII being 0.5 in green algae [67].

Y_II_ and ETR_in situ_ were quantified at different hours of local time (9:00, 14:00 and 17:00) and in different days from 8 random algal samples of the reactor.

(b) Ex situ—for rapid light curve measurements, 3 replicates were taken from raceway ponds, transported to the laboratory and introduced in 50 mL tubes covered by dark foil. Firstly, algae were incubated 15 min in darkness and basal fluorescence was determined by switching on measured light (F_o_), and then saturation light pulse was applied to measure maximal Florescence (F_m_). Maximal quantum yield (F_v_/F_m_) was determined as:

F_v_/F_m_ = F_m_ − F_o_/F_m_ (7)

Then algal samples were exposed for 30 s to twelve increasing irradiances (25, 45, 66, 90, 125, 190, 285, 420, 625, 845,1150, and 1500 μmol photons m^−2^ s^−1^) of actinic red light followed by a saturating light pulse determining effective quantum yield (Y_II_) and ETR as it is indicated above Equations (5) and (6). This ETR is denominated ex situ ETR. ETR versus irradiance obtained from light curves were fitted according to [71] models to estimate the variables of maximal electron transport rate, i.e., ETR_max_, photosynthetic efficiency (α_ETR_) and saturated irradiance (E_k_).

The non-photochemical quenching of fluorescence (NPQ) is calculated as follows:

NPQ = (F_m_ − F’_m_)/F’_m_ (8)

NPQ max is calculated by the fitting of NPQ versus irradiance function by using [71] model.

NPQ can also be expressed as the ratio Y_NPQ_/Y_NO,_ according to [66]. Y_NO_ is the fraction of energy passively dissipated as heat and fluorescence, mainly due to closed PSII reaction centres. High values indicate an inability of the macroalga to protect itself against photodamage by an excess of radiation. Y_NPQ_ is the fraction of energy dissipated as heat via regulated photoprotective mechanisms. High values are indicative of photoprotective capacity. The ratio ETR_max_/NPQ_max_ represents an estimator of the ratio between productivity and energy dissipation, according to [67].

### 4.5. Statistical Analysis

Statistical analyses were performed using Statgraphics Centurion 19.3.02 software. One-way ANOVAs were performed for photosynthetic variables in both trials (density and acclimatization) after verification of the fulfilment for data normal distribution and homogeneity of variance. Otherwise, a non-parametric Kruskal-Wallis analysis was done. For means separation after ANOVAs, Tukey’s test was used. Pearson correlation between response variables was performed using Statistica 7 software.

## 5. Conclusions 

As observed in this study, raceway pond photobioreactors can be used for biomass production of *Ulva compressa* in recirculating conditions. The increase of biomass productivity at the highest algal density was related to the increase of photosynthetic capacity.

Optimal biomass productivity was 0.32–0.36 g FW L^−1^ day^−1^ (6–8 g DW m^−2^ day^−1^). These values are in the range of microalgal biomass productivity in this type of photobioreactor [73], by agriculture fertilizers were added at very lower amounts than in other studies [26], simulating the nutrient range reached by using fishpond effluents [62,63,74]. In spite of this, we consider that there is a limitation of nutrients in our culture and the increase of nutrients would allow us to increase biomass production. The best concentration of biomass for culture in circumstances similar to ours could be 0.8–1.0 kg FW m^−2^, since they produce same or even higher amount of biomass and can compete better with the microalgae that may appear in the reactor when it is open air and water is recirculated continuously. It could probably improve if the amount of water is increased in the raceway pond to 20 cm depth (200 L m^−2^) or even 30 cm (300 L m^−2^) and the nutrient concentration is increased, which would result in higher self-shading, less temperature variability during the day and less nutrient availability.

*Ulva* biomass produced in raceways could be used to extract bioactive compounds and is suitable for use in feed due to its good protein content (13.7–38.9% protein) [5,62,75,76], which allows this industry to be more sustainable and resilient as the market demands [77,78,79]. This also applies to the manufacture of biofertilizers and biostimulants using algae as raw material [80,81,82]. In addition, *Ulva* in raceway systems due to the high nutrient consumption can supply an ecosystem service related to nutrient biofiltration. *Ulva* strains have been used for bioremediation and water purification systems for both industry, farms and urban settings [83,84,85] and it can reach a similar biofiltration capacity to microalgae [86,87,88], but with a cheaper harvesting process since it is not necessary to conduct flocculation or centrifugation, making this species a source of low-cost compounds. In addition, aquaculture of highly productive *Ulva* strains could contribute to the mitigation of climate change, although there is controversy about the actual contribution of carbon sink and sequestration [89,90].

More biotechnological research is necessary to get sustainable cultures of *Ulva* strains, reducing the cost in the frame of the Blue economy.

## Figures and Tables

**Table 1:** Growth parameters of *Ulva compressa* at different culture densities, determined at the end of every weekly experiment.

Culture Density	SGR (% Day^−1^)	Biomass Production (kg FW)	Growth Rate (g FW m^−2^ Day^−1^)	Growth Rate (g DW m^−2^ Day^−2^)	Biomass Production (kgFW/mg N)	Biomass Production (kg FW/mg P)
0.6 kg m^−2^	5.94	7.7	36.7	6.23	0.611	8.280
0.8 kg m^−2^	4.9	8.2	38.1	6.48	0.651	8.817
1.0 kg m^−2^	4.07	8.3	39.1	6.64	0.659	8.925

**Table 2:** Photosynthetic parameters of Ulva compressa form Rapid Light Curves determined by using a Mini PAM fluorometer. Different letters correspond to significant differences between treatments following one-way ANOVA (*p* < 0.05) after Tukey’s test.

Culture Density	F_v_/F_m_	ETR_max_ (µmol m^−2^ s^−1^)	α_ETR_ (µmol Electrons/µmol Photons)	E_k_ (µmol Photons m^−2^ s^−1^)	NPQ_max_	ETR_max_/NPQ_max_
0.6 kg m^−2^	0.62 ± 0.010 ^B^	89.02 ± 12.16 ^A^	0.22 ± 0.008 ^A^	412.00 ± 44.79 ^B^	0.74 ± 0.074 ^C^	120.01 ± 15.34 ^A^
0.8 kg m^−2^	0.63 ± 0.010 ^B^	90.51 ± 11.62 ^A^	0.22 ± 0.034 ^A^	352.81 ± 15.23 ^B^	1.41 ± 0.044 ^B^	64.31 ± 27.03 ^B^
1.0 kg m^−2^	0.68 ± 0.007 ^A^	94.82 ± 5.69 ^A^	0.18 ± 0.009 ^A^	512.46 ± 36.22 ^A^	1.51 ± 0.028 ^A^	62.69 ± 4.79 ^B^

**Table 3:** Growth parameters of Ulva compressa acclimatized during 1 week at the photobioreactor conditions, determined at the end of the experiment.

Culture Density	SGR (% Day^−1^)	Biomass Production (kg FW)	Growth Rate (g FW m^−2^ Day^−1^)	Growth Rate (g DW m^−2^ Day^−1^)	Biomass Production (kg FW/mg N)
Pre−acclimatized	4.07	8.30	38.10	6.48	0.66
Post−acclimatized	4.80	10.1	47.62	8.10	0.79

**Table 4:** Photosynthetic parameters of U. compressa derived from RLCs performed on acclimated algae and non-acclimated algae at 1.0 kg FW m^−2^ density. Different letters correspond to significant differences between treatments following S-tudent’s *t*-test (*p* < 0.05).

Algae Condition	F_v_/F_m_	ETR_max_ (µmol m^−2^ s^−1^)	α_ETR_ (µmol Electrons /µmol Photons)	E_k_ (µmol Photons m^−2^ s^−1^)	NPQ_max_	ETR_max_/NPQ_max_ (µmol m^−2^ s^−1^)
Pre−acclimation	0.680 ± 0.007 ^B^	94.89 ± 5.69 ^B^	0.180 ± 0.009 ^B^	512.460 ± 36.221 ^B^	1.510 ± 0.028 ^A^	62.69 ± 4.79 ^B^
Post−acclimation	0.740 ± 0.006 ^A^	184.73 ± 28.52 ^A^	0.230 ± 0.009 ^A^	852.340 ± 99.315 ^A^	0.750 ± 0.027 ^B^	245.98 ± 45.85 ^A^

**Table 5:** Cultivation conditions and biomass yields of *Ulva* spp. cultivated in different experimental systems. Modified from [26].

Species	Tank Volume (L)	Stocking Density (kg FW m^−2^)	Growth (g L^−1^ Day^−1^)	Water Exchange (L Day^−1^)	Growth Rate (g DW m^−2^ Day^−1^)	References
*U. compressa*	3000	0, 6–1	0.37–0.48	0	6,23–8	This study
*U. pseudorotundata*	200	1.2	Not	0	7.5–8	[6]
*U. lactuca*	800	1–3	0.32–0.17	0	25–13	[26]
*U. rigida*	110	1.9	Not	2, 4–96	44–73	[42]
*U. rigida*	1900	1.9	Not	14, 4	48	[43]
*U. lactuca*	600	1	0.19–0.63	34	11–38	[44]
*U. reticulata*	40	1	1.35–2.3	2040	46	[45]
*U. rigida*	750	2.5	0.09–0.32	2–12	40	[46]
*U. lactuca*	600	2–6	0.24–0.42	4–16	55	[47]
*U. lactuca*	600	1	Not	4–8	55	[48]
*U. lactuca*	1700	1	Not	1–24	45–16	[49]
*U. lactuca*	600	1–8	0.37–0.16	12	12, 32	[50]
*U. lactuca*	600	1.5	0.39	2	21, 3	[51]
*U. lactuca*	900–1700	1	0.26–0.64	14–56	19	[21]

**Table 6:** Values of Pearson correlations values among the following parameters: SGR (%), ETR_max_, ETR_max_/NPQ and Nitrate Uptake Rate (NUR). Asterisk indicates positive significant values (*p* > 0.01).

	SGR (%)	ETRmax (µmol m^−2^ s^−1^)	ETRmax/NPQmax (µmol m^−2^ s^−1^)	NUR (µmol N g^−1^ DW h^−1^)
SGR (%)	−	−0.2689	0.8147 *	0.9969 *
ETRmax	−	−	0.0772	−0.2605
ETRmax/NPQmax	−	−	−	0.8353 *

## Data Availability

No new data were created or analyzed in this study. Data sharing is not applicable to this article.
